# Effects of Adding *Clostridium* sp. WJ06 on Intestinal Morphology and Microbial Diversity of Growing Pigs Fed with Natural Deoxynivalenol Contaminated Wheat

**DOI:** 10.3390/toxins9120383

**Published:** 2017-11-27

**Authors:** FuChang Li, JinQuan Wang, LiBo Huang, HongJu Chen, ChunYang Wang

**Affiliations:** 1 Shandong Provincial Key Laboratory of Animal Biotechnology and Disease Control and Prevention, Shandong Agricultural University, 61 Daizong Street, Taian City 271018, China; chlf@sdau.edu.cn (F.L.); huanglibo123@126.com (L.H.); hjchen72@sdau.edu.cn (H.C.); 2 Feed Research Institute, Chinese Academy of Agricultural Sciences, Beijing 100081, China; wangjinquan@caas.net.cn

**Keywords:** *Clostridium* sp. WJ06, deoxynivalenol, pig, intestinal morphology, microbial diversity

## Abstract

Deoxynivalenol (DON) is commonly detected in cereals, and is a threat to human and animal health. The effects of microbiological detoxification are now being widely studied. A total of 24 pigs (over four months) were randomly divided into three treatments. Treatment A was fed with a basal diet as the control group. Treatment B was fed with naturally DON-contaminated wheat as a negative control group. Treatment C was fed with a contaminated diet that also had *Clostridium* sp. WJ06, which was used as a detoxicant. Growth performance, relative organ weight, intestinal morphology, and the intestinal flora of bacteria and fungi were examined. The results showed that after consuming a DON-contaminated diet, the growth performance of the pigs decreased significantly (*p* < 0.05), the relative organ weight of the liver and kidney increased significantly (*p* < 0.05), and the integrity of the intestinal barrier was also impaired, though the toxic effects of the contaminated diets on growing pigs were relieved after adding *Clostridium* sp. WJ06. The data from MiSeq sequencing of the 16S ribosomal ribonucleic acid (rRNA) gene and internal transcribed spacer 1 (ITS1) gene suggested that the abundance of intestinal flora was significantly different across the three treatments. In conclusion, the application of *Clostridium* sp. WJ06 can reduce the toxic effects of DON and adjust the intestinal microecosystem of growing pigs.

## 1. Introduction

Deoxynivalenol (DON), also known as vomitoxin, is synthesized mainly by the toxigenic fungi of the *Fusarium* genus, which is commonly detected in cereals, particularly wheat, barley, maize, and their by-products all over the world [1]. This mycotoxin remains stable for many years when stored at room temperature, or even when heated at 135 °C. Its deactivation occurs through the destruction of the epoxide ring under drastic acid or alkaline conditions, reactions with aluminum and lithium hydrates or peroxides, and hydration in autoclave [2]. Therefore, DON readily enters the human and animal food chains [3,4,5]. DON causes toxic effects in humans as well as in all animal species investigated to date. Among animal species, pigs show a relatively high sensitivity to DON because of the high percentage of cereals in their diet and the lack of microorganisms in the front of their small intestine, which are able to degrade mycotoxins before DON is absorbed by the small intestine [6,7].

The intestinal tract is the first target for mycotoxins following ingestion of contaminated feed. Consumption of DON-contaminated feed in pigs impacts the gastrointestinal tract, causing epithelial injuries of the stomach and the intestine, and leading to intestinal inflammatory response [8,9]. In vitro and in vivo studies have also demonstrated that DON inhibits intestinal nutrient absorption, alters intestinal cell functions, and compromises the intestinal barrier [10]. Additionally, stability of the intestinal flora appeared to be an important factor in animal health. Surprisingly, the effect of DON on intestinal microflora has been poorly investigated [11]. Direct impact of DON on intestinal microflora composition has never been reported, and only few data are available for other members of the trichothecene toxin group [12,13].

Due to the detrimental effects of mycotoxins, some strategies have been developed to prevent the growth of mycotoxigenic fungi and also to decontaminate and detoxify foods and feeds [14]. These strategies include: (1) the prevention of mycotoxin contamination; (2) the detoxification of mycotoxins present in foods and feeds; and (3) the inhibition of mycotoxin absorption in the gastrointestinal tract. Although numerous physical and chemical detoxification methods have been tested, none really provide the necessary efficacy and safety [15]. Since cost-effective methods to detoxify mycotoxin-contaminated grains and foods are urgently needed to minimize potential losses to the farmer and toxicological hazards to the consumer, it is a necessity to find new suitable methods for the decontamination of mycotoxins. Therefore, the development of microbiological detoxification measures is essential for improving the safety of these foods for human consumption. Microbes or their enzymes could be used for mycotoxin detoxification, and such biological approaches are now being widely studied [16,17]. DON, as well as other trichothecenes, may have its chemical structure altered by bacteria or fungi, which utilize their enzymatic systems as a carbon source.

Therefore, the present study aims to: (i) investigate the influence of naturally DON-contaminated wheat on the intestinal morphology and intestinal microflora of growing pigs; and (ii) evaluate the effect of *Clostridium* sp. WJ06 on the intestinal morphology and intestinal microflora of growing pigs as a detoxicant.

## 2. Results

### 2.1. Effect of Clostridium sp. WJ06 on Growth Performance and Relative Organ Weight of Growing Pigs

The effect of *Clostridium* sp. WJ06 and DON-contaminated wheat on the growth performance of growing pigs is shown in Table 1. In this study, average daily body weight gain (ADG) and average daily feed issue (ADFI) decreased significantly more than in the control group after feeding DON-contaminated wheat (*p* < 0.05), while the ratio of feed intake to body weight gain (F/G) increased significantly (*p* < 0.05). There were no significant differences (*p* ≥ 0.05) from the control group after adding *Clostridium* sp. WJ06.

**Figure 1:**
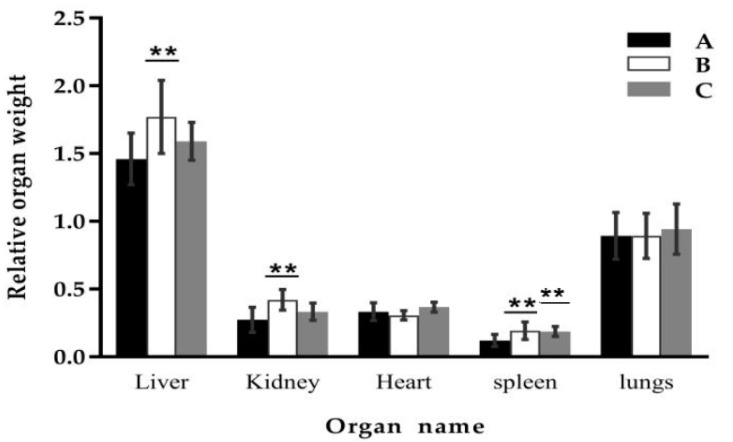
Effect of *Clostridium* sp. WJ06 on relative organ weight ^1^ of growing pigs. ^1^ Relative organ weight (kg/kg) = organ weight (kg)/live weight (kg). A, B, and C represent the samples of different treatments. Bars are presented as mean ± SD, *n* = 3. ** Indicates that results differ significantly between treatments (*p* < 0.05).

The effect of *Clostridium* sp. WJ06 on the relative organ weight of growing pigs is shown in Figure 1. The results showed that the relative weights of the liver, kidney, and spleen of growing pigs increased significantly more than in the control group after feeding DON-contaminated wheat (*p* < 0.05), while there were no significant differences (*p* ≥ 0.05) for the relative weights of the heart and lungs. After adding *Clostridium* sp. WJ06, the relative weight of the liver and kidney were not significantly different (*p* ≥ 0.05) to the control group, while the spleen increased significantly (*p* < 0.05).

### 2.2. Effects of Clostridium sp. WJ06 on Intestinal Morphology of Growing Pigs

**Figure 2:**
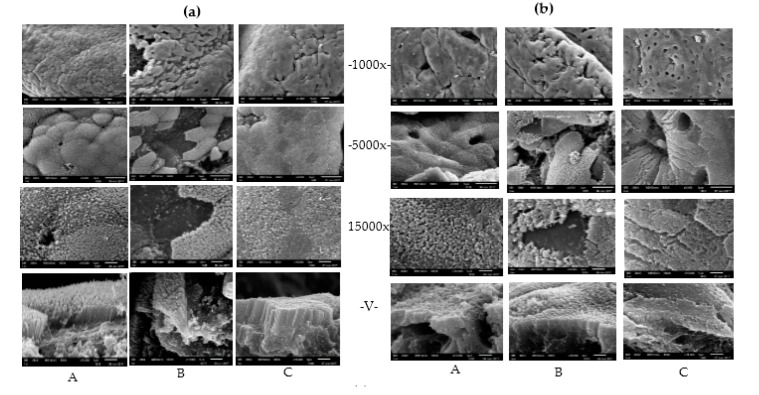

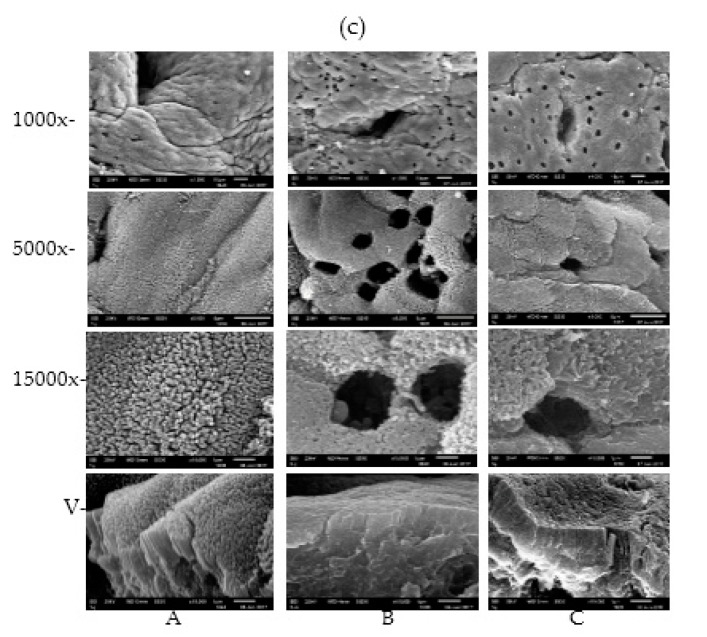
The effect of *Clostridium* sp. WJ06 on the morphology of different intestine regions of growing pigs via scanning electron microscopy (*n* = 3). (**a**–**c**) refer to samples of the ileum, caecum, and colon, respectively. A, B, and C refer to the samples of different treatments. 1000×, 5000×, and 15,000× represent the magnification of electron microscopy at transverse sections. V represents the photo of a vertical section magnified 15,000×.

Representative morphologies of the ileum, caecum and colon are illustrated in Figure 2. Scanning electron microscope observations revealed that the mucosa of ileum, caecum and colon were severely damaged after pigs were fed with DON-contaminated wheat compared with the control group, especially for ileum. Additionally, epithelial cells on the surface of the intestinal villus were not integrated and exhibited histological lesions. In contrast, the damage to the villus barriers of the intestine from contaminated diets were relieved significantly after adding *Clostridium* sp. WJ06 to the diets of pigs. 

### 2.3. Analysis of Operational Taxonomic Units (OTUs) and the Alpha Diversity of Bacteria Flora and Fungal Flora in all Samples

**Figure 3:**
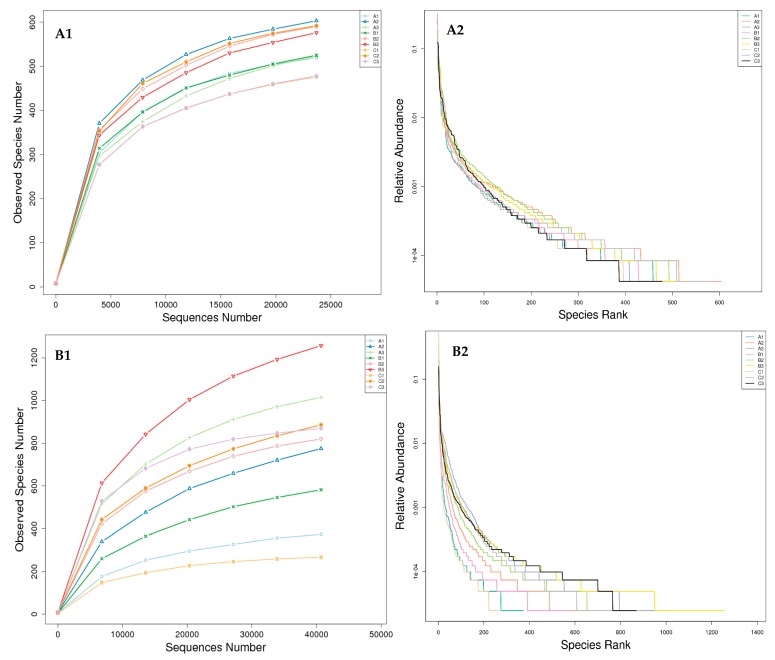
Rarefaction curve and rank abundance curve in nine libraries. (**A1**,**A2**) represented the data from 16S ribosomal ribonucleic acid (rRNA) gene sequencing. (**B1**,**B2**) represented the data from internal transcribed spacer 1 (ITS1) gene sequencing. In the rarefaction curves plot (**A1** & **B1**), the *x*-axis is number sequencing reads randomly chosen from a certain sample to obtain operational taxonomic units (OTUs). The *y*-axis is corresponding OTUs. In the rank-abundance curves plot (**A2** & **B2**), the *x*-axis is the abundance rank, and the *y*-axis is the relative abundance. The higher the abundance, the smaller the rank. Curves for different samples are represented by different colors.

Alpha diversity was applied in analyzing the complexity of species diversity for one sample, and two indices (rarefaction curves and rank-abundance curves) were selected to identify community richness and diversity (Figure 3). Rarefaction curves reflect the reasonability of the number of sequencing reads used for analysis, and can be used to infer species richness in the sample. A flat curve means that the number of sequencing reads is reasonable, and less new species can be detected with increasing sequencing reads. Rank abundance curves directly reflect the richness and evenness of species in the sample. Species richness can be viewed as the range of the curve in the horizontal direction. The results demonstrated that the curves of all of the samples have almost approached the saturation plateau, which indicates that the 16s ribosomal ribonucleic acid (rRNA) gene and the internal transcribed spacer 1 (ITS1) gene sequence database was very abundant, and the current analysis had adequate depth to capture most microbial diversity information.

In the present study, a total number of 253,708 qualified sequence reads were obtained from nine samples via V3–V4 16S rRNA sequencing, with an average of 43,177 effective sequence reads for each sample (the minimum sample was 34,392, and the maximum was 47,673), and the average length of the effective bacterial sequence reads was 389 bp (Table 2). At the same time, the data of the operational taxonomic units (OTUs) and the richness estimator by ITS1 sequencing are listed in Table 3. A total number of 605,933 qualified sequence reads were obtained from nine samples, with an average of 67,326 effective sequence reads for each sample (the minimum sample was 46,157, and the maximum was 102,600), and the average length of the effective fungal sequence reads was 227 bp (Table 3).

**Figure 4:**
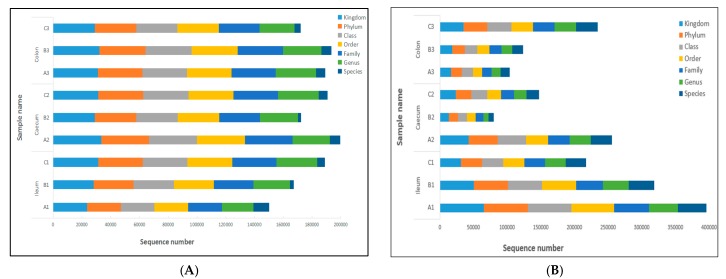
Tag abundance of each sample at different classification levels. Sequence number indicates the number of sequences annotated to that level, which are expressed in different colors. Bars are presented as means, *n* = 3. (**A**) The sequence number of bacteria flora in different treatments. (**B**) The sequence number of fungi flora.

In order to analyze the species diversity within samples, we clustered all of the effective tags to operational taxonomic units (OTUs) at 97% similarity. Then, we performed species annotation based on the OTUs’ representative tags. According to the results of species annotation, the statistics of the sequence numbers in different classification levels (kingdom, phylum, class, order, family, genus, and species) are calculated and displayed in Figure 4. The composition of each sample and differences among samples can be understood through Figure 4. The results in our study showed that the sequence number of fungi flora varied significantly in the three treatments at several classification levels, while that of bacteria flora were not shifted significantly; that is, the structure of intestinal bacteria flora were relatively simple and stable.

### 2.4. Effects of Clostridium sp. WJ06 on Intestinal Bacterial Flora of Pigs via 16S rRNA Gene Sequencing

**Figure 5:**
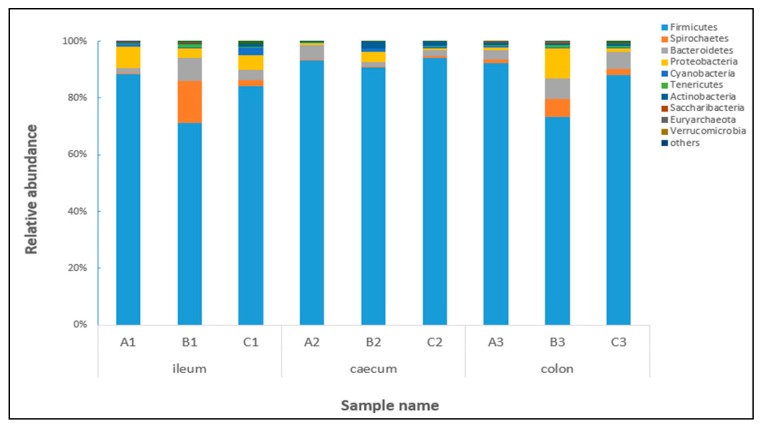
Relative abundance of the dominant bacterial phyla level in nine libraries. Each bar represents the relative abundance of each sample. Each color represents a particular bacterial family. Sequences that could not be classified into the top 10 were classified as ‘others’. A, B, and C represent the samples from three treatments, and 1, 2, and 3 represent the samples of the ileum, caecum and colon, respectively.

**Figure 6:**
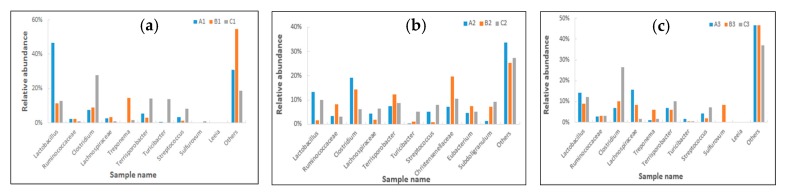
Relative abundance of the dominant bacteria at the same gut region at the genus level. Each bar represents the relative abundance of each sample. Each color represents a particular bacterial family. (**a**) A1, B1, and C1 represent the samples of the ileum in different treatments. (**b**) A2, B2, and C2 represent the samples of the caecum in different treatments. (**c**) A3, B3, and C3 represent the samples of the colon in different treatments.

In this paper, the top 10 species of bacteria flora with the highest relative abundance at the phylum and genus levels are shown in Figure 5 and Figure 6, respectively. Based on relative abundance, *Firmicutes*, *Spirochaetes*, *Bacteroidetes*, and *Proteobacteria* were identified as the major bacterial taxa of the pig bacterial community in all of the samples. For *Firmicutes* in particular, the average relative abundance was over 86% in different treatments. As shown in Figure 5, the abundance of *Firmicutes* in the ileum and colon decreased significantly after being fed with contaminated diets, while that of *Spirochaetes* and *Bacteroidetes* in the ileum and colon were significantly increased. However, the abundance of these bacteria was not affected after adding *Clostridium* sp. WJ06. The result also showed that the dominant bacteria of caecum were not significantly affected by contaminated diets.

*Lactobacillus* represented the largest number of bacteria in the ileum (47%), ceacum (13%), and colon (14%) at the genus level, especially for ileum. The abundance of *Lactobacillus* was significantly decreased in the ileum (11%), ceacum (2%) and colon (9%) after being fed with contaminated diets, while that of *Lactobacillus* was higher than the control group in the ceacum (10%) and colon (12%), but not in the ileum (13%) after adding *Clostridium* sp. WJ06 (Figure 6). Meanwhile, the abundance of *Clostridium* increased significantly after the use of *Clostridium* sp. WJ06. The abundance of *Clostridium* in the ileum and colon of the control treatment were all 7%, while that of *Clostridium* in the ileum and colon increased to 28% and 27%, respectively, after adding *Clostridium* sp. WJ06. The abundance of *Clostridium* in the ceacum of the control treatment was 19%, but fell to 6% after adding *Clostridium* sp. WJ06. However, the abundance of *Clostridium* was not affected by contaminated diets, which may be connected with the reproductive ability of *Clostridium* sp. WJ06 and was different in the ileum, caecum, and colon, respectively.

**Figure 7:**
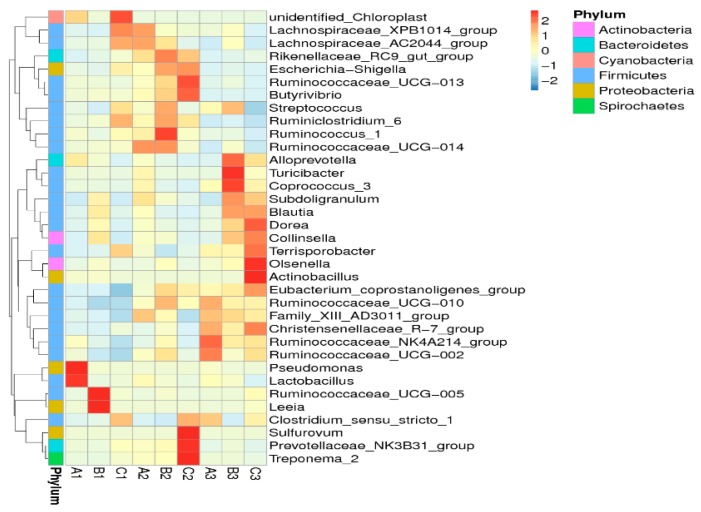
Hierarchically clustered heat map of the highly represented fungal taxa (at the genus level) in nine libraries. The relative percentages (%) of the bacterial families are indicated by varying color intensities, according to the legend at the top of the figure. The darker the color of samples, the higher the relative abundance shown in the picture.

In our study, the result showed that the structure of porcine cecal flora was quite different from that of the flora of the ileum and colon. C*hristensenellaceae*, *Eubacterium*, and *Subdoligranulum* belong to the dominant flora in the ceacum, but not in the colon and ileum. At the same time, *Sulfurovum*, *Leeia*, and *Treponema* formed dominant populations in the ileum and colon, but not in the ceacum. The shifts of the bacterial community compositions were further corroborated by a clear clustering of the dominant bacterial genus corresponding to different treatment in the heat map, as shown in Figure 7. The result revealed that the bacterial composition in the ileum, ceacum, and colon of growing pigs varied significantly in different diets, but the structure of bacteria was relatively stable and simple.

### 2.5. Effects of Clostridium sp. WJ06 on Intestinal Bacterial Flora of Pigs via ITS1 Gene Sequence

**Figure 8:**
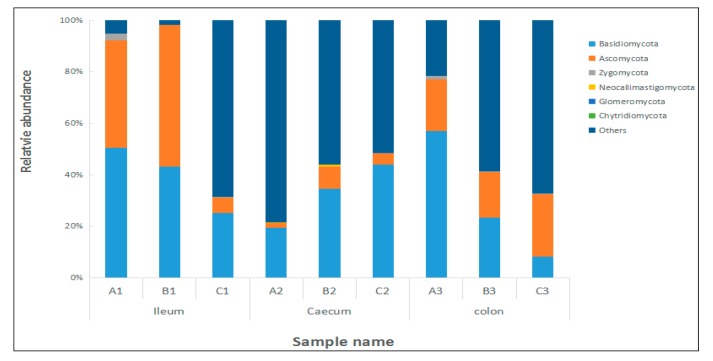
Dominant fungi composition of the different communities at the phyla level. Each bar represents the relative abundance of each sample. Each color represents a particular bacterial family. Sequences that could not be classified into top 10 were assigned as ‘others’. A, B, and C represent the samples from three treatments, and 1, 2, and 3 represent the samples of the ileum, caecum, and colon, respectively.

**Figure 9:**
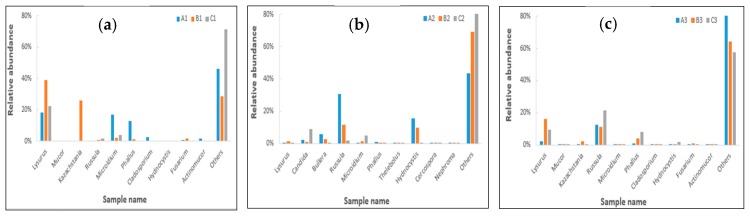
Relative abundance of the dominant fungi at the same gut region at the genus level. Each bar represents the relative abundance of each sample. Each color represents a particular bacterial family. (**a**) A1, B1, and C1 represent the samples of ileum in different treatments. (**b**) A2, B2, and C2 represent the samples of caecum in different treatments. (**c**) A3, B3, and C3 represent the samples of the colon in different treatments.

In this paper, the top 10 species of fungal flora with the highest relative abundance at the phylum and genus levels are shown in Figure 8 and Figure 9, respectively. It can be seen from Figure 8 that the number of dominant fungi species varied significantly in the ileum, caecum, and colon in different diets, especially in regard to the abundance of *Basidiomycota* and *Ascomycota*.

As shown in Figure 9, the results revealed that the abundance of *Lysurus, Kazachstania*, and *Fusarium* were significantly increased in the ileum and colon at the genus level after being fed with contaminated diets, and that these bacteria had not significantly increased after adding *Clostridium* sp. WJ06. The result indicated that the species structure of the caecum was significantly different from that of the ileum and colon.

**Figure 10:**
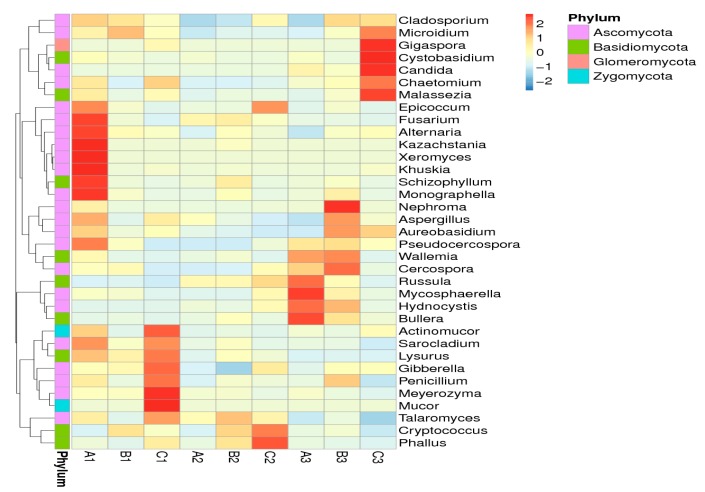
Hierarchically clustered heat map of the highly represented fungal taxa (at the genus level) in nine samples. The relative percentages (%) of the bacterial families are indicated by varying color intensities according to the legend at the top of the figure. The more brunette the samples’ color, the higher relative abundance shown in the picture.

The shifts of the fungal community compositions were further corroborated by clear clustering of the dominant bacterial genus corresponding to different treatments in the heat map, as shown in Figure 10. The results suggested that the number of fungi in the treatment groups varied greatly.

### 2.6. Analysis of Beta Diversity of all Samples

**Figure 11:**
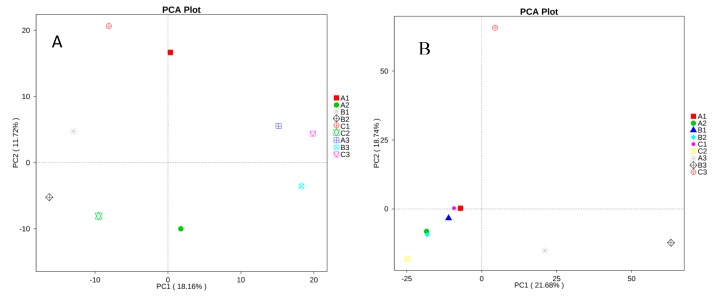
Principal component analysis (PCA) in nine libraries. Scatterplot of PCA score depicting the variance in fingerprints derived from different bacterial communities. The more similar the composition of samples, the closer the distance shown in the PCA picture. (**A**) The sequence number of bacteria flora. (**B**) The sequence number of fungi flora.

Beta diversity analysis was used to evaluate the differences of samples in species complexity, and principal component analysis (PCA) was carried out for this paper [18]. The PCA plot based on the relative abundance of bacterial OTUs revealed a separation between the different treatments and different intestinal regions on the basis of the first two PC scores, which accounted for 11.72% and 18.16% of the total variation, respectively (Figure 11A). The more similar the composition of samples, the closer the distance shown in the PCA picture. Our data showed that the bacterial flora diversity varied significantly in the different intestinal regions, but there was no significant difference in the different treatments.

At the same time, the fungal diversity was analyzed by PCA, and the two PC scores accounted for 18.74% and 21.68% of the total variation, respectively (Figure 11B). Our data showed that the fungal flora diversity varied significantly more in the colon than in the ileum and caecum in different diets.

### 2.7. Analysis of the Concentration of DON and De-Epoxy-DON (DOM-1) in Urine and Feces

**Figure 12:**
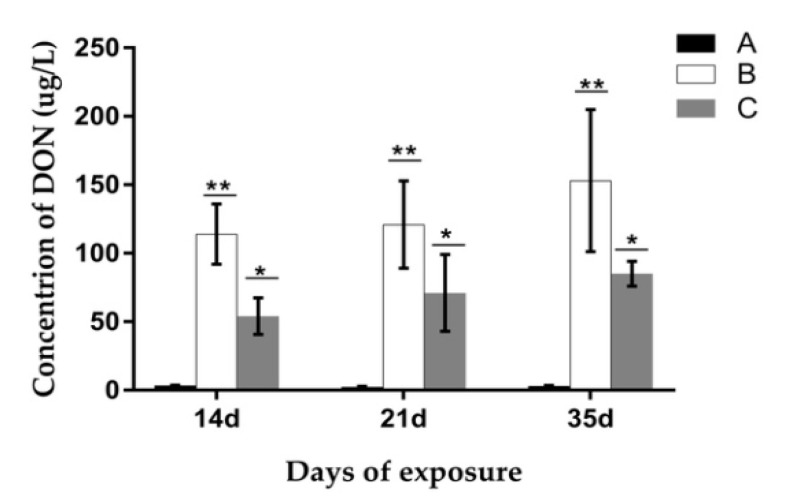
Concentration of deoxynivalenol (DON) in porcine urine at different exposure days. A, B, and C represent the samples of different treatments. Bars are presented as mean ± SD, *n* = 5. The values with different asterisks (* or **) differed significantly on the same exposure day (*p* < 0.05).

De-epoxydeoxynivalenol (DOM-1) had not been found in porcine urine in any experimental group. The concentration of DON in urine at different trail periods is displayed in Figure 12. The result indicated that on the 14th, 21st, and 35th day of the experiment, the DON value in group B was significantly higher (*p* < 0.05) when compared with the other groups. Meanwhile, the DON value in group C was significantly lower when compared to group B (*p* < 0.05), but significantly higher than the control group (*p* < 0.05). No significant differences were found in each group on different exposure days.

**Figure 13:**
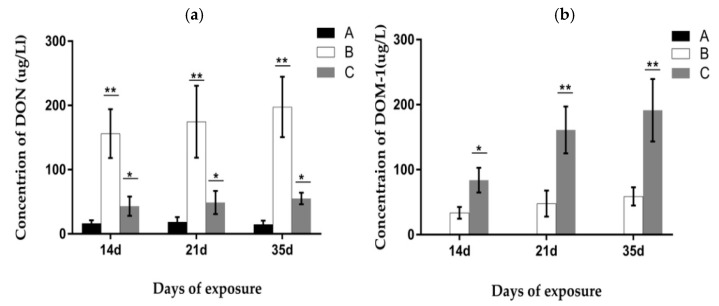
Concentration of DON and de-epoxy-DON (DOM-1) in porcine feces at different exposure days. (**a**) The concentration of DON. (**b**) The concentration of DOM-1. A, B, and C represent the samples of different treatments. Bars are presented as mean ± SD, *n* = 5. The values with different asterisks (* or **) in the same exposure day differ significantly (*p* < 0.05).

The concentration of DON and DOM-1 in porcine feces is displayed in Figure 13. The result showed that the DON value in group B increased significantly compared with the other groups (*p* < 0.05), and a gradual increasing trend of the DON value from day 14 to day 35 appeared in groups B and C. The concentration of DOM-1 had not been found in group A in our study. The DOM-1 value of group B decreased significantly more than that of group C (*p* < 0.05), while the values at days 21 and 35 were significantly higher than the value at day 14 (*p* < 0.05). This result indicated that DOM-1 was mainly converted in the intestinal tract by *Clostridium* sp. WJ06.

## 3. Discussion

### 3.1. DON Metabolism and the Toxic Effects of DON

**Figure 14:**
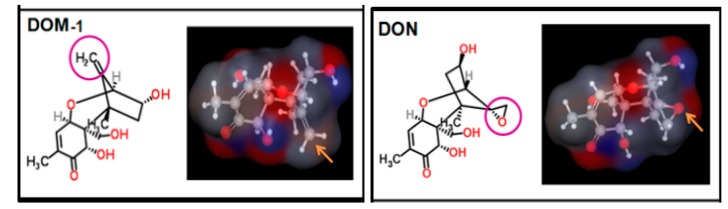
Chemical structure of DON and DOM-1 was cited by Maresca [24]. DON and DOM-1 were drawn using Marvin software. Images on the right show an electrostatic map of the molecules, with the blue color indicating a positive region, the red color indicating a negative region, and the gray color indicating a neutral region. The purple circles on the left images and the yellow arrows on the right images indicate the position of the epoxide or de-epoxide functions in DON and DOM-1, respectively.

Since the discrepancy existed in the absorption, metabolism and clearance procedures, the tolerance for the toxicity and pathogenic dose of DON were different for different animal species in vivo [19,20]. DON was degraded by microorganisms into a variety of products (such as de-epoxy-DON) in the digestive tract or in the intestinal mucosa, liver, kidney, and other organs. Chemical structure of DON and DOM-1 was listed in Figure 14. Residual DON and DOM-1 were the highest in the pig kidney, followed by the liver, and the high DON residue in the kidney may be associated with urine enrichment [21,22,23].

In this study, the relative weight of the liver and kidney of growing pigs increased significantly after being fed with DON-contaminated wheat, indicating that the toxicity of DON was related to its metabolism and residual presence in the liver and kidney, which was consistent with the result reported before [21,22,23]. Meanwhile, the toxin effects on the liver and kidney of growing pigs were relieved after adding *Clostridium* sp. WJ06. These results come from the analysis of DON and DOM-1 in porcine feces and urine, and revealed that DOM-1 was mainly converted in the intestinal tract by *Clostridium* sp. WJ06. This indicated that the detoxified effect of *Clostridium* sp. WJ06 may be because it can convert DON to DOM-1 in the intestinal tract, and then reduce the absorption of DON in the intestine.

The results of this study also showed that DON-contaminated wheat can also cause a significant increase in the relative weight of the spleen, which was inconsistent with previous results [21,22]. *Clostridium* sp. WJ06 had no protective effect on the spleen of pigs, which may be due to the coexistence of other mycotoxins in the naturally contaminated wheat. The special metabolic pathways may exist in the spleen, so that *Clostridium* sp. WJ06 cannot decompose the toxicity of a variety of mycotoxins on the spleen of pigs.

### 3.2. Intestinal Morphological Structural Integrity and Toxic Effects of DON

The integrity of the intestinal barrier can effectively prevent intestinal bacteria, toxins, inflammatory mediators, and other harmful substances from passing through the intestinal mucosa into the blood [12,25]. DON is mainly absorbed in the small intestine, and it can pass through the intestinal barrier, enter the blood circulation and distribution system, and travel to the peripheral organs, thereby affecting the activity and function of the cells [26,27,28]. The absorption rate of DON exhibited the great difference between animal species; for example, the absorption rates of pigs, chickens, sheep, and cows were 82%, 19%, 9.9%, and 1%, respectively [29,30]. These differences are mainly related to the distribution of parasitic flora before or after the small intestine [31]. Before entering the small intestine of ruminants and poultry, DON is exposed to higher concentrations of microorganisms, which can convert DON to DOM-1 [32]. For humans and monogastric animals, there are a lack of microorganisms in the front of the small intestine, so humans and pigs are more sensitive to DON than poultry and ruminants [33,34].

In this study, the results suggested the microstructure of the ileum, caecum, and colon were destroyed by DON, especially ileum, which may be due to fewer microorganisms in the ileum of pigs than the caecum and colon. After adding *Clostridium* sp. WJ06, the damage to the intestinal barrier were alleviated significantly. The results indicated that DON may induce intestinal lesions by disrupting the integrity of the intestinal barrier, which is consistent with previous findings [35]. *Clostridium* sp. WJ06’s protection of the intestine is probably because it can convert DON to DOM-1 in the intestine, thereby reducing its absorption and protecting the intestinal barrier of growing pigs. In the future, a study concerning the mechanism of *Clostridium* sp. WJ06 on degrading DON needs to be carried out.

### 3.3. Effects of Clostridium sp. WJ06 on Intestinal Microflora Diversity

The animal digestive tract, especially that of mammals, is very suitable for microbial mass reproduction. The complex microbial communities that survive in the intestine are called microbial flora [36]. Intestinal microflora planted in the small intestine and large intestine play an important role in maintaining the host’s functions, including the energy intake of food, generation of the host’s key metabolites, development of the immune system, response to gastrointestinal diseases, and so on [37]. In this research, the effects of *Clostridium* sp. and DON on the diversity of bacteria and fungi in the pig intestine were studied by MiSeq sequencing of the 16S rRNA gene and the ITS1 gene.

At the phylum level, *Firmicutes* is the predominant group of bacteria in the intestinal tract of mammals, with a proportion of more than 80%. *Bacteroidetes* is in the intestinal tract of second heterotic groups, which are connected with fermentation of carbohydrates, glucose metabolism, and bile acid metabolism. In the analysis of the genus level, *Lactobacillus* was the largest number of bacteria in the ileum, caecum and colon, especially in the ileum. The abundance of *Lactobacillus* was significantly decreased in all of the intestinal regions. A number of physiological influences of *Lactobacillus* on their hosts have been examined, including antimicrobial effects, microbial interference, supplementary effects on nutrition, antitumor effects, the reduction of cholesterol in serum, and immunomodulatory effects, and so on [38,39]. The analysis concerning the diversity of fungal flora of growing pigs in different diets was listed in this paper.

In conclusion, the results indicated that the bacterial and fungal composition in the ileum, caecum and colon of growing pigs varied significantly in different diets, but the structure of bacteria was relatively stable and simple. The effect of detoxification of *Clostridium* sp. WJ06 may be connected with the adjustment effect on the composition and diversity of intestinal microflora. Meanwhile, our results suggested that the composition and structure of the caecum of pigs was different significantly compared with that of the ileum and colon.

## 4. Materials and Methods

### 4.1. Ethics Statement

All procedures were carried out in accordance with the Guidelines for Experimental Animals of the Ministry of Science and Technology (Beijing, China) for the ethical use of experimental animals. The protocol were reviewed and approved by the Animal Care and Use Committee in the Shandong Agricultural University of China. (Permit Number ACSA-2016-036, 15 September 2016).

### 4.2. Bacteria Culture

**Figure 15:**
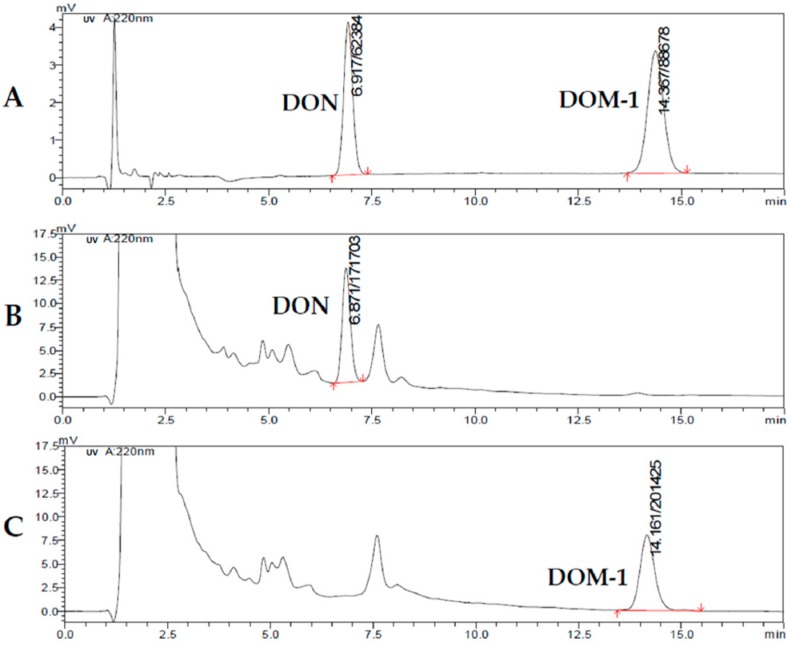
The effect of *Clostridium* sp. WJ06 culture on degrading DON in vitro via high performance liquid chromatography (HPLC). (**A**) The chromatogram of standard DON and DOM-1. (**B**) The chromatogram of the co-culture of DON (20 ppm) and L10. (**C**) The chromatogram of the co-culture of DON (20 ppm), L10, and *Clostridium* sp. WJ06.

*Clostridium* sp. WJ06 (Patent No: CN102485883.B) was separated from the intestinal tract of adult chickens, provided by Dr. Wang Jinquan of the Chinese Academy of Agricultural Sciences. L10 broth or agar was used to culture *Clostridium* sp. WJ06 at 37 °C for 48–72 h in an anaerobic chamber with an atmosphere of 85% N_2_, 10% CO_2_, and 5% H_2_. At the same time, the conversion of DON was determined by high performance liquid chromatography (HPLC), and the results showed that the DON can be converted into DOM-1 with a degradation rate of over 90% by *Clostridium* sp. WJ06 (Figure 15) and has good stability.

### 4.3. Diets, Animals, and Experimental Design

The basal diet on the basis of de Blas and Wiseman (1998) and the addition of DON-contaminated wheat diets (33.3%) were completed before the trial of the seventh day, and the diet composition and nutrition level are shown in Table 4. DON, Aflatoxin B1 (AFB1), Fumonisin B1 (FB1) and Zearalenone (ZEA) were all examined by LC-MS/MS in the Institute of Quality Standards and Detection Technology, Chinese Academy of Agricultural Sciences (Beijing, China).

Twenty-four commodity generation PIC pigs (quinary crossbred Pig improve Co. England, four months of age) of both sexes (equal male to female ratio per treatment and 55.58 ± 1.83 kg in mass) were used for experiments after an adaptation period of seven days. The pigs were divided into eight healthy pigs per treatment group and assigned to the three experimental diets by average liver mass, which was monitored every day. Treatment A was only fed with basal diet as a control group. Treatment B was fed with the basal diet with the addition of DON-contaminated wheat as the negative control group. Treatment C was fed with the basal diet with the addition of DON-contaminated wheat, but using *Clostridium* sp. WJ06 as a detoxicant every day. The addition amount of *Clostridium* sp. was 30 mL per pig every day, and the culture solution of *Clostridium* sp. WJ06 was poured into the feed and mixed completely before feeding in the morning. The feeding experiment was carried out at a Kongjiazhuang farm in Shandong province.

The experiment lasted for 35 days, including a seven-day adaptation period and a 28-day experimental period. Feeds and water were provided ad libitum and offered at 8:30 am and 5:30 pm daily. The residual feed was collected daily. Pigs were weighed individually at the beginning and the end of the experiment, and the average daily weight gain (ADG) was calculated. The average daily feed intake (ADFI) was recorded, and the feed intake to body weight gain (F/G) ratio was calculated.

On days 14, 21, and 35 of the experiment, the urine and feces of 20 pigs (five pigs per treatment) were collected and stored at −20 °C for further analysis. On the 35th day of the experiment, three healthy pigs randomly selected from each treatment group were euthanized by electric shock at the same time (8:00 am). The liver, kidney, heart, and other organs were separated and weighed. The relative weight of the organ (kg/kg) = organ weight (kg)/live weight (kg) was calculated. Meanwhile, their digestive tracts had been removed within less than 15 min. The content of the intestines (including the ileum, caecum, and colon) were collected and frozen immediately in liquid nitrogen, and subsequently stored at −70 °C until analyzed. Then, the approximately two-centimeter segment of the middle portion of the ileum, caecum, and colon was fixed in 2.5% glutaraldehyde at room temperature for observation under a scanning electron microscope.

### 4.4. DNA Extraction

Twenty-seven samples (200 mg of the intestinal content of the ileum, caecum, and colon) were suspended in 1.4 mL of ASL buffer (stool lysis buffer, Qiagen, Hilden, Germany), and DNA was extracted using a QIAamp DNA Stool Mini kit (Qiagen, Hilden, Germany), following the manufacturer’s instructions. The bacterial cells were disrupted by a bead beater with sterile zirconia beads added to the samples, which improved extraction yield and the quality of the community DNA [40]. For each sample, DNA was extracted in duplicate to avoid bias [41], and the extracts from the same sample were pooled for 16S rRNA sequencing. To assess the DNA quality, A 260/280 measurements were performed using a DU640 Nucleic Acids and Protein Analyzer (Beckman Coulter, Brea, CA, USA). The DNA samples were diluted to 20 ng/uL using sterile ultrapure water. After the extracted DNA was detected and quantified strictly, equal moles of three DNA samples of the same treatment and the intestinal regions were mixed [42]. Therefore, the total number of mixed DNA samples was nine, and then nine libraries were coded as A1, A2, A3, B1, B2, B3, C1, C2, and C3; that is, A, B, and C represented the samples from three treatments, and 1, 2, and 3 represented the samples of the ileum, caecum, and colon, respectively. Then, all of the samples were stored at −20 °C until use.

### 4.5. MiSeq Sequencing of 16S rRNA Gene and ITS1 Gene

For all of the samples collected, we used a barcoded high-throughput sequencing approach similar to that described in Jia et al. [43] and McGuire et al. [44] to survey the diversity and composition of the bacterial and fungal species in each of these samples. PCR amplifications were conducted with the barcoded primer pair 341F/806R set that amplifies the V3–V4 fragments of the 16S rRNA gene (341F: CCTAYGGGRBGCASCAG, 806: GGACTACNNGGGTATCTAAT). Meanwhile, the first internal transcribed spacer region (ITS1) of the fungal rRNA gene was amplified using the ITS5-1737F (GGAAGTAAAAGTCGTAACAAGG) and ITS2-2043R (GCTGCGTTCTTCATCGATGC) as the primer pair. All of the amplicon sequencing samples were accomplished using the Illumina MiSeq platform at the Novogene Bioinformatics Technology Co., Ltd., Beijing, China.

At first, all of the initial template DNA concentrations were normalized between samples. PCR reactions were performed according to protocols described by Caporaso et al. [45]. All of the PCR reactions were carried out with Phusion^®^ High-Fidelity PCR Master Mix (NEB, Beijing, China).

We mixed the same volume of 1× loading buffer (contained SYB green) with PCR products and operate electrophoresis on 2% agarose gel for detection. Samples with a bright main strip between 400–450 bp were chosen for further experiments. PCR products were mixed in equidensity ratios. Then, the mixture of PCR products was purified with a Qiagen Gel Extraction Kit (Qiagen, Hilden, Germany). Sequencing libraries were generated using a TruSeq^®^ DNA PCR-Free Sample Preparation Kit (Illumina, San Diego, CA, USA) following the manufacturer’s recommendations, and index codes were added. The library quality was assessed on the Qubit^®^ 2.0 Fluorometer (Thermo Scientific, Waltham, MA, USA) and Agilent Bioanalyzer 2100 system (Agilent, Waldbronn, Germany). At last, the library was sequenced on an IlluminaHiSeq2500 platform and 250 bp paired-end reads were generated.

### 4.6. Bioinformatics Analysis

Paired-end reads were assigned to each sample based on their unique barcode and truncated by cutting off the barcode and primer sequence. The high-quality clean tags were obtained, which complied with the method reported by Jia et al. [43]. These sequences were classified into the same OTUs (operational taxonomic units) at an identity threshold of 97% similarity using UPARSE software (UPARSE v7.0.1001, Edgar, Tiburon, California, USA, http://drive5.com/uparse/) [46]. For each representative sequence, the GreenGene Database (http://greengenes.lbl.gov/cgi-bin/nph-index.cgi) was used based on the Ribosomal Database Project (RDP) classifier (Version 2.2, http://sourceforge.net/projects/rdp-classifier/) algorithm to annotate taxonomic information. The taxon abundance of each sample was generated into phylum, class, order, family, and genera levels. In order to study the phylogenetic relationship of different OTUs, and the difference of the dominant species in different samples (groups), multiple sequence alignments were conducted using the MUSCLE software (Version 3.8.31, http://www.drive5.com/muscle/) [47].

We processed the ITS1 fungal amplicon data following methods outlined in McGuire et al. [47]. We used the QIIME pipeline in a similar way as for the 16S rRNA data, except the Unite Database (https://unite.ut.ee/) was used based on the Blast algorithm, which was calculated by QIIME software (Version 1.9.1, http://qiime.org/scripts/assign_taxonomy.html) to annotate taxonomic information. 

All of the analyses from clustering to alpha diversity (within each sample) and beta diversity (between samples) were performed with QIIME (Version 1.9.10,) and displayed with *R* software (Version 2.15.3) [48].

### 4.7. Analysis of the Concentration of DON and DOM-1 in Urine and Feces

The concentrations of DON and de-epoxy-DON (DOM-1) in urine and feces were analyzed by LC-MS/MS (liquid chromatography-tandem mass spectrometry). The sample preparation methods were described in detail earlier [49]. LC-MS/MS analysis was performed using an Agilent 1200 liquid chromatograph (Agilent Technologies, Palo Alto, CA, USA) coupled to a 3200 QTrap^®^ mass spectrometry system (Applied Biosystems, Foster City, CA, USA) equipped with a Turbo electrospray ionization (ESI) interface. All investigations concerning the concentration of DON and DOM-1 were carried out in the Institute of Quality Standards and Detection Technology, Chinese Academy of Agricultural Sciences.

### 4.8. Statistical Analysis

The data analyses for growth performance and the relative weight of organs were performed using one-way analysis with SPSS for Windows, version 14.0, and differences between means were compared by Duncan’s least significant difference. *p* value < 0.05 was considered a significant difference, and the analysis result was noted with mean ± standard deviation (mean ± SD).

## Figures and Tables

**Table 1:** Effect of *Clostridium* sp. WJ06 on the growth performance of pigs.

Treatment *	Initial Weight (kg)	Final Weight (kg)	ADG (g) ^#^	ADFI (kg) ^#^	F/G ^#^
A	54.38 ± 1.21	78.3 ± 1.78	885.93±72	2.14±0.38	2.41 ± 0.32
B	56.44 ± 1.63	71.33 ± 2.98 ^a^	551.48±46 ^a^	1.66±0.33 ^a^	3.01 ± 0.41 ^a^
C	56.06 ± 1.78	76.07 ± 3.15	741.16 ± 62	1.98 ± 0.27	2.68 ± 0.36

Values are presented as Mean ± SD, *n* = 8. The values with different small letters ^(a)^ in the same column differ significantly (*p* < 0.05). ^#^ ADG, average daily body weight gain; ADFI, average daily feed intake; F/G, the ratio of feed intake to body weight gain, indicating feeding efficiency. Feed was calculated based on dry weight. * Treatment A refers to the control diet; Treatment B refers to the contaminated diet; Treatment C refers to the contaminated diet with *Clostridium* sp. WJ06 added.

**Table 2:** OTUs ^1^ data and richness estimator of nine libraries via 16S rRNA gene sequencing.

Sample Name *	Raw PE ^#^	Qualified ^#^	Base (nt) ^#^	AvgLen (nt) ^#^	Effective% ^#^	Chao Ave ^2^	Observed Species	Shannon Ave ^2^
A1	64,782	34,392	10,231,113	390	40.5	564.27	426	4.93
B1	69,691	41,156	14,360,310	388	53.05	496.63	380	4.66
C1	68,531	40,201	13,531,583	384	51.41	538.44	443	5.44
A2	72,117	47,250	15,233,409	389	54.37	434.19	365	5.21
B2	64,769	45,377	14,399,002	389	57.1	436.26	389	5.90
C2	69,357	45,332	14,537,019	385	54.4	379.02	329	5.53
A3	75,203	47,673	14,834,644	392	50.28	489.40	406	4.85
B3	68,288	41,553	14,224,802	396	52.59	468.35	382	5.05
C3	70,715	45,665	14,628,909	386	53.59	586.57	485	6.00
Total	409,247	253,708	82,292,436	2325	311	4393	3605	48
Average	69,272	43,177	13,997,865	389	51.92	488.13	401	5.29

* A1, B1, and C1 represent the sequences of ileum from different treatments. A2, B2, and C2 represent the sequences of caecum from different treatments. A3, B3, and C3 represent the sequences of colon from different treatments. ^1^ The operational taxonomic units (OTUs) were defined at 3% dissimilarity level. ^2^ The richness estimators (Chao) and diversity indices (Shannon) were calculated using the program QIIME. ^#^ Raw PE means original PE reads tested by the Illumina MiSeq platform. Qualified means the raw reads with chimeric sequences and low quality sequences removed. Base refers to the final effective data of the basic group. AveLen refers to the average length of effective tags. Effective means Effective Tags/Raw PE × 100%.

**Table 3:** OTUs ^1^ data and richness estimator of nine libraries via ITS1 gene sequencing.

Sample Name *	Raw PE ^#^	Qualified ^#^	Base (nt) ^#^	AvgLen (nt)^#^	Effective% ^#^	Chao Ave ^2^	Observed Species	Shannon Ave ^2^
A1	123,899	72,335	18,813,512	267	56.92	798.77	611	5.18
B1	70,194	54,056	12,893,920	246	74.80	389.73	287	2.92
C1	121,183	102,600	22,150,221	218	83.97	656.80	447	3.46
A2	103,737	79,891	17,630,954	221	76.95	843.35	570	5.07
B2	72,542	46,157	10,630,776	234	62.49	992.45	667	5.71
C2	95,418	78,657	16,739,208	213	82.33	656.80	665	3.46
A3	78,988	64,817	12,797,508	198	81.95	959.79	748	6.87
B3	83,591	62,692	13,180,068	211	74.72	1195.65	918	6.52
C3	72,307	44,728	10,577,687	238	61.34	886.02	769	5.96
Total	821,859	605,933	135,413,854	2046	655.47	7379.36	5681	45.14
Average	91,318	67,326	15,045,984	227	72.83	819.93	631	5.02

* A1, B1, and C1 represent the sequences of ileum from different treatments. A2, B2, and C2 represent the sequences of caecum from different treatments. A3, B3, and C3 represent the sequences of colon from different treatments. ^1^ The operational taxonomic units (OTUs) were defined at 3% dissimilarity level. ^2^ The richness estimators (Chao) and diversity indices (Shannon) were calculated using the program QIIME. ^#^ Raw PE means original PE reads tested by the Illumina MiSeq platform. Qualified means the raw reads with chimeric sequences and low quality sequences removed. Base refers to the final effective data of the basic group. AveLen refers to the average length of effective tags. Effective means Effective Tags/Raw PE × 100%.

**Table 4:** Composition of the experimental diets.

	Basal Diet	Contaminated Diet
Ingredients (%)		
Corn	60.0	32.7
Soybean Meal	23.0	18.0
Extruded soybean	2.0	1.0
Contaminated wheat	0	33.3
Concentrate feed ^#^	15	15
Total	100	100
Calculated composition (%)		
Dry matter	85.4	86.62
Crude ash	4.79	4.88
Calcium	0.63	0.58
Available phosphorus	0.57	0.62
Crude fibre	53.75	52.55
Crude protein	16.67	16.70
Crude fat	3.78	2.72
Digestible energy (MJ/kg)	17.17	16.69
Content of mycotoxin (ug/kg) ^2^		
DON	371.2	1904.44
Aflatoxin B1 (AFB1)	1.56	1.59
Fumonisin B1 (FB1)	256.3	325.7
Zearalenone (ZEA)	145.16	96.08

^#^ Concentrate feed provided per kg diet: Ca 2.1 g; P 1.2 g; Mn 6 mg; Fe 150 mg; Zn 150 mg; Cu 9 mg; I 0.21 mg; Se 0.45 mg; vitamin A 3300 IU; vitamin D 290 IU; vitamin E 24 IU; vitamin K 30.75 mg; vitamin B1 1.50 mg; vitamin B2 5.25 mg; vitamin B6 2.25 mg; vitamin B 120.026 mg; pantothenic acid 15 mg; nicotinic acid 22.50 mg. ^2^ Measured by HPLC methods.
